# The Addition of a Synthetic LPS-Targeting Domain Improves Serum Stability While Maintaining Antimicrobial, Antibiofilm, and Cell Stimulating Properties of an Antimicrobial Peptide

**DOI:** 10.3390/biom10071014

**Published:** 2020-07-08

**Authors:** Anna Maystrenko, Yulong Feng, Nadeem Akhtar, Julang Li

**Affiliations:** Animal Biosciences, University of Guelph, Guelph, ON N1G 2W1, Canada; gmaystre@uoguelph.ca (A.M.); yfeng09@uoguelph.ca (Y.F.); akhtarn@uoguelph.ca (N.A.)

**Keywords:** antimicrobial peptide, protegrin-1, stability, hybridization, biofilm, serum

## Abstract

Multi-drug resistant (MDR) bacteria and their biofilms are a concern in veterinary and human medicine. Protegrin-1 (PG-1), a potent antimicrobial peptide (AMP) with antimicrobial and immunomodulatory properties, is considered a potential alternative for conventional antibiotics. AMPs are less stable and lose activity in the presence of physiological fluids, such as serum. To improve stability of PG-1, a hybrid peptide, SynPG-1, was designed. The antimicrobial and antibiofilm properties of PG-1 and the PG-1 hybrid against MDR pathogens was analyzed, and activity after incubation with physiological fluids was compared. The effects of these peptides on the IPEC-J2 cell line was also investigated. While PG-1 maintained some activity in 25% serum for 2 h, SynPG-1 was able to retain activity in the same condition for up to 24 h, representing a 12-fold increase in stability. Both peptides had some antibiofilm activity against *Escherichia coli* and *Salmonella typhimurium*. While both peptides prevented biofilm formation of methicillin-resistant *Staphylococcus aureus* (MRSA), neither could destroy MRSA’s pre-formed biofilms. Both peptides maintained activity after incubation with trypsin and porcine gastric fluid, but not intestinal fluid, and stimulated IPEC-J2 cell migration. These findings suggest that SynPG-1 has much better serum stability while maintaining the same antimicrobial potency as PG-1.

## 1. Introduction

Biofilms are multilayer communities of immobile bacteria that grow adhered to a surface, encased within a self-produced extracellular polymeric substance (EPS) matrix, and differ from their planktonic counterparts growing in nutrient-rich media [1]. These bacterial communities can be found growing on both organic and inorganic surfaces. Biofilms growing on inorganic surfaces are most often associated with hospital-acquired pathogens growing within medical devices, such as cardiac pacemakers and other medical implants [2]. However, bacterial biofilms have been isolated from a variety of surfaces, including water lines and feeding troughs used for animals, as well as equipment and surfaces within meat processing plants, serving as sources for continual infection and posing the risk of spreading zoonotic disease [3]. Meanwhile, biofilms growing on organic surfaces are associated with chronic infections of epithelial and mucosal surfaces, such as *Pseudomonas*-associated cystic fibrosis [4], urinary tract infections, and foot ulcers [5]. In animals, these infections manifest as mastitis, wound lesions, enteritis, and pneumonia [6]. Treatment for biofilm infections is scarce as many biofilms have an inherent resistance to conventional antibiotics due to the protective EPS matrix and can persist even after treatment with concentrations a thousand times their minimal inhibitory concentration [7].

Antimicrobial peptides (AMPs) are a promising area of therapeutic agents with great potential as an alternative for conventional antibacterial therapies. Many have noted the potential application of AMPs and their synthetic analogues as antibiofilm agents [8,9,10,11,12,13,14,15]. These small cationic and amphipathic peptides function as the first line of defense for a variety of animals and plants and, as such, play a key role in both antimicrobial activity and immunomodulation. The cathelicidin family of peptides is one such group of AMPs [16]. Human cathelicidin, LL-37, has been reported to act as a chemoattractant for neutrophils, monocytes, and T cells [17] and induces chemokine and cytokine production [18]. Porcine cathelicidin protegrin-1 (PG-1) was shown to modulate immune activity through the stimulation and cell migration in porcine epithelial cells through the activation of an insulin-like growth factor 1 receptor [19]. Meanwhile, human beta-defensin 2 can inhibit the classical pathway through C1q binding, reducing the over-activation of the complement cascade during thermal injury [20].

Despite their strong antimicrobial and immunomodulatory activities, clinical application of AMPs has been slow, in part due to the challenges with poor drug delivery as a result of instability within physiological fluids. The myriad of proteases and inhibiting factors present in both the gastrointestinal tract, as well as within the circulatory system, are major contributors to reducing peptide activity [21,22,23,24]. Due to the high salt concentration of serum, positively charged ions interfere with the electrostatic attraction of AMPs to the bacterial surface by masking the negative charge [25]. Meanwhile, the fluctuation of digestive fluid pH within the digestive tract may destabilize peptide bonds, decreasing their activity [21,23]. Chemical modification of peptides, such as incorporation of D-amino acids, cyclization, acetylation, and amidation of N- and C- termini, has been shown to improve their structural stability [24,26,27,28,29]. However, such strategies for improvement result in costly production [30]. It is also possible that modification of the peptide’s sequence may abolish its immunomodulatory properties, along with mechanisms that may hold greater importance than a peptide’s immediate antimicrobial activity [31].

In contrast to chemical modification, hybridization of unmodified amino acid sequences has been demonstrated as an effective technique for improving AMPs without jeopardizing their potential for recombinant production in a biological expression system [32,33,34]. However, this strategy for peptide improvement has often been studied with the aim of increasing antimicrobial activity versus stability. In the interest of investigating the method of hybridization as a means to improve AMPs activity and stability in physiological fluids, we hybridized PG-1 with a synthetic lipopolysaccharide (LPS) binding sequence, previously designed by Kim et al. [35]. This design was chosen amongst two other LPS-binding domains studied by Kim et al., including a truncated Lactoferrin (Lf) and a bactericidal/permeability-increasing (BPI) protein sequence. After the candidate sequences were screened in a pilot study for stability-improving effects (unpublished), the “Syn” sequence was chosen as the most promising and was selected for the remainder of the study. The aim of the current study is to compare the original unmodified peptide to its synthetic hybrid in three different aspects: the stability within physiological fluids; the activity against planktonic growth and biofilm formation of multi-drug resistant pathogens; and the impact on intestinal epithelial cells.

## 2. Materials and Methods

### 2.1. Materials

#### 2.1.1. Peptide Synthesis and Preparation 

Standard lyophilized PG-1 and SynPG-1 peptides were synthesized at approximately 80% purity (Top-Peptide Co; Shanghai, China), dissolved in filter-sterilized acidified water (0.01% acetic acid), and diluted to 20 mg/mL stock solutions. Due to the amphipathic nature of the peptides, acidified dissolvent is necessary, otherwise there is a risk of peptide precipitation. Peptide standards were stored at −80 °C. 

#### 2.1.2. Bacterial Strains and Culture Preparation

Veterinary clinical isolates, including *Salmonella typhimurium,* multi-drug resistant *Escherichia coli*, methicillin-resistant *Staphylococcus aureus* (MRSA), and methicillin-resistant *Staphylococcus pseudointermedius* (MRSP), were kindly provided by Dr. H. Cai (Department of Pathobiology, University of Guelph, ON, Canada). An antibiogram for these strains is available in the supplementary text (Table S1). Gram-negative strains were cultured in Luria−Bertani (LB) broth. Gram-positive strains were cultured in Tryptic soy broth (TSB). Both cultures were grown under aerobic conditions at 37 °C.

### 2.2. 3D Structural Modeling 

Iterative threading ASSEmbly refinement (I-TASSER) was used to validate the 3D structure of SynPG-1 [36,37,38]. The Nuclear Magnetic Resonance defined structure for Protegrin-1 (PDB ID: 1PG1), deduced by Fahrner et al., was used to create the 3D model of PG-1 [39]. Molecular graphics and analyses were performed with UCSF ChimeraX, developed by the Resource for Biocomputing, Visualization, and Informatics at the University of California, San Francisco, USA, with support from National Institutes of Health R01-GM129325, the Office of Cyber Infrastructure and Computational Biology, and the National Institute of Allergy and Infectious Diseases [40].

### 2.3. Antimicrobial Activity on Panktonic Cell Growth

The minimum bactericidal concentration (MBC) of SynPG-1 against the tested pathogens was identified through a broth macrodilution antimicrobial assay as previously described, with some modification [41]. Briefly, mid-logarithmic phase cells were diluted to a concentration of about 1 × 10^5^ CFU/mL, and 20 µL of diluted cell suspension was added to a 20 mM sodium phosphate buffer (pH 7) of up to 480 µL for a final volume of 0.5 mL. The buffer volume was adjusted to accommodate the addition of SynPG-1 and PG-1 so that the final solution volume was 0.5 mL for each tested concentration. Final peptide concentrations ranged from 0.1 µg/mL to 8 µg/mL. PG-1 at the known MBC (2 µg/mL *E. coli*; 4 µg/mL *S. typhimurium*; 0.5 µg/mL MRSP; 0.5 µg/mL MRSA) was considered the in-house positive control, while the suspension containing no peptide was considered the negative control. Experimental and control tubes were incubated for 2 h at 37 °C without shaking to allow for bactericidal action to occur. After incubation, experimental and control tubes were serially diluted to 1:100 in appropriate growth media and 100 µL were spread on an appropriate nutrient agar plate using 4–6 sterile glass beads per plate. Plates were left at 37 °C for 18 h to allow colonies sufficient time to reach an easily observable size. All concentrations of peptide were tested in three independent experiments. Colony numbers from each plate were recorded. The MBC was defined as the concentration that killed 99.9% of all colonies, as compared to the negative control. 

### 2.4. Funcitonal Antimicrobial Peptide Stability 

#### 2.4.1. Activity After Incubation in Serum

To compare serum stability, a similar procedure was utilized, as described in Dong et al. [42], with some modification. Briefly, a solution of 25% pig serum was made by diluting fresh pig serum with phosphate buffer saline (PBS). SynPG-1 and PG-1 were added for a final concentration of 40 µg/mL of peptide in the 25% pig serum solution. Suspension containing no peptide was considered the negative control. Experimental and control tubes were incubated for up to 7 or 24 h in 37 °C to allow for serum constituent interaction with the peptide. At designated time points, 100 µL of peptide and serum suspension was removed and heat deactivated for 35 min at 56 °C. After heat deactivation and a 5 min cooling period, 100 µL of the antimicrobial peptide suspension was added to a solution containing 8 µL of mid-logarithmic phase *E. coli* cells, diluted to a concentration of about 1 × 10^5^ CFU/mL and 92 µL of 20 mM sodium phosphate buffer (pH 7) for a total volume of 200 µL. Experimental and control tubes were incubated for 2 h at 37 °C without shaking to allow for bactericidal action to occur. Peptides were tested at a concentration 10 times their MBC to ensure that observed differences in antimicrobial activity were due to the impact of serum and not a variation in peptide activity. The remaining procedures conducted were as per the antimicrobial assay described earlier (Section 2.3). This experiment was carried out three independent times. Serum stability was evaluated as the relative colony growth on plates from bacterial grown with serum and peptide treatments, in comparison to plates grown from bacteria grown with serum alone. Diluted serum did not impact bacterial growth.

#### 2.4.2. Activity After Incubation with the Trypsin Enzyme

Procedures similar to those used by Carmona et al. [43] were used for this experiment. SynPG-1 and PG-1 were tested at molar ratios of 100:1 and 20:1 of peptide to trypsin (0.5 g porcine Trypsin-EDTA Solution, Sigma-Aldrich, Oakville, ON, Canada) in digestion buffer (50 mM Tris buffer pH 7.5, Oakville, ON, Canada) at a final solution volume of 500 µL. Suspension containing no peptide was considered the negative control. Experimental and control tubes were incubated for up to 4 h in 37 °C without shaking to allow for enzyme digestion with the peptide. At designated time points, 100 µL of peptide and trypsin suspension was removed and heat deactivated for 5 min at 70 °C (BioRad Thermocycler, Mississauga, ON, Canada). After heat deactivation and a 5 min cooling period, 100 µL of the antimicrobial peptide suspension was added to a solution, containing 8 µL of mid-logarithmic phase *E. coli* cells diluted to a concentration of about 1 × 10^5^ CFU/mL and 92 µL of 20 mM Sodium phosphate buffer (pH 7) for a total volume of 200 µL. Peptides were tested at a concentration 10 times their MBC to ensure that the observed differences in antimicrobial activity were due to the impact of the trypsin enzyme and not a variation in peptide activity. The remaining procedures conducted were as per the antimicrobial assay described earlier (Section 2.3). This experiment was carried out three independent times. Trypsin stability was evaluated as the relative colony growth on plates from bacteria with trypsin and peptide treatments, in comparison to plates grown from bacteria with trypsin alone. Diluted trypsin did not appear to impact bacterial growth. 

#### 2.4.3. Collection of Porcine Digestive Fluids

Porcine digestive fluids were collected, as previously described by Wang et al. [44]. Briefly, the porcine gastrointestinal tract was obtained from the Meat Science Laboratory (University of Guelph, Guelph, ON, Canada), courtesy of Brian McDougall. The gastrointestinal tract was kept at 4 °C during dissection, and collection of contents from the stomach and upper small intestine (duodenum and jejunum) was conducted within 5 h of sacrifice. Contents were centrifuged at 10,000 rpm (9600× *g*) for 10 min at 4 °C, and supernatant fluid was collected and stored at −80 °C until its use in the stability experiment. The pH of the collected supernatants were 3.5 and 6.5 for gastric fluid and duodenum fluid, respectively. 

#### 2.4.4. Activity After Incubation in Porcine Digestive Fluids 

Procedures similar to those used by Egger et al. [45] were used for this experiment. SynPG-1 and PG-1 were incubated at 37 °C with shaking in a PBS solution consisting of 25% or 50% digestive fluid for 1 h at a final concentration of 1 mg/mL. Suspension containing no peptide was considered the negative control. After incubation, 10 µL of the incubated solutions was added for a total volume of 0.5 mL, consisting of 20 µL of mid-logarithmic phase *E. coli* cells, diluted to a concentration of about 1 × 10^5^ CFU/mL, and 470 µL of 20 mM sodium phosphate buffer (pH 7). Peptides were tested at a concentration 10 times their MBC to ensure that observed differences in antimicrobial activity were due to the impact of digestive fluid and not a variation in peptide activity. The remaining procedures conducted were as per the antimicrobial assay described earlier (Section 2.3). This experiment was carried out three independent times. Stability in porcine digestive fluid was evaluated as the relative colony growth on plates from bacterial grown with digestive fluid and peptide treatments, in comparison to plates grown from bacteria grown with digestive fluid alone. Diluted digestive fluids marginally impacted bacterial growth but did not yield significantly lower Colony Forming Units (CFU) when compared to bacteria grown without the addition of diluted digestive fluids. 

### 2.5. Assessment of Biofilim Inhibition and Destruction

#### 2.5.1. Biofilm Inhibition Assay

To test the inhibition of biofilm formation, in a 96-well plate, 15 µL of overnight culture of either *E. coli*, *S. typhimurium*, or MRSA were added to 150 µL of minimal salt media (40 mM NaOH ∙ 7H_2_O; 22 mM KH_2_PO_4_; 18.68 M NH_4_CL; 740 mM NaCl; 2 mM MgSO_4_; 0.1 mM CaCl_2_; 22 mM supplemented with d-Glucose), treated with PG-1 or SynPG-1. Similar to two previous studies, tested concentrations ranged from ½× MBC to 32× MBC, depending on the strain, with 6 replicates for each well [46,47]. Plates were placed in a static incubator at 37 °C for 48 h; the time required for untreated wells to form biofilm. After incubation, treated media was removed and wells were rinsed with 155 µL of PBS to remove planktonic cells. After sufficient air drying of wells, 150 µL of 0.1% Crystal violet stain was added to each well and incubated at room temperature for 30 min, after which, dye was removed and wells were rinsed again with 155 µL of PBS. After sufficient drying of wells, 150 µL of 70% ethanol solution was added to each well and incubated at room temperature for 30 min. After 30 min, 100 µL of each well was added to a new 96-well plate and absorbance was measured at an OD of 590 nm. 

#### 2.5.2. Pre-Formed Biofilm Destruction Assay

To test the destruction of preformed biofilms, in a 96-well plate, 15 µL of overnight culture, of either *E. coli*, *S. typhimurium*, or MRSA, was added to 150 µL of minimal salt media (40 mM NaOH ∙ 7H_2_O; 22 mM KH_2_PO_4_; 18.68 M NH_4_CL; 740 mM NaCl; 2 mM MgSO_4_; 0.1 mM CaCl_2_; 22 mM supplemented with d-Glucose) in each well. Plates were placed in a static incubator at 37 °C for 48 h; the time required for untreated wells to form biofilm. After formation, minimal salt media was removed and wells were treated with 155 µL of minimal salt media containing PG-1 or SynPG-1. Similar to two previous studies, tested concentrations ranged from ½× MBC to 32× MBC, depending on the strain, with 6 replicates for each well [46,47]. Plates were placed in a static incubator at 37 °C for 3 h. After 3 h, treated media was removed and wells were rinsed with 155 µL of PBS to remove planktonic cells. After sufficient drying of wells, 150 µL of 0.1% crystal violet stain was added to each well and incubated at room temperature for 30 min, after which dye was removed and wells were rinsed again with 155 µL of PBS. After sufficient drying of wells, 150 µL of 70% ethanol solution was added to each well and incubated at room temperature for 30 min. After 30 min, 100 µL of each well was added to a new 96-well plate and absorbance was measured at an OD of 590 nm. 

#### 2.5.3. Enumeration of Viable Cells after Biofilm Inhibition

In order to investigate whether prevention or degradation of biofilm by peptide treatment impacted bacterial cell growth within the biofilm, an enumeration assay was carried out. The following protocol was developed using methods described in the work by Wilson et al. [48]. Briefly, in separate 15 mL culture tubes, 20 µL of overnight culture of either *E. coli*, *S. typhimurium*, or MRSA were added to 1 mL of minimal salt media (40 mM NaOH ∙ 7H_2_O; 22 mM KH_2_PO_4_; 18.68 M NH_4_CL; 740 mM NaCl; 2 mM MgSO_4_; 0.1 mM CaCl_2_; 22 mM supplemented with d-Glucose) and treated with PG-1 or SynPG-1. Due to the laborious procedure, the study was limited to measuring cell viability for only two concentrations, basing the choice on the highest significant reduction of biomass from crystal violet staining for each strain. For instance, for MRSA, significant biomass reduction was seen at 8 µg/mL and 16 µg/mL. These concentrations were chosen for the enumeration assay. In instances where no substantial reduction was seen, as in the case of *E. coli*, the highest tested biomass concentration, or higher, was chosen to confirm the lack of activity on viability. In the case of *S. typhimurium*, initial enumeration at 1 µg/mL and 2 µg/mL showed limited impact on viability despite significant reduction in biomass staining, thus two higher concentrations (4 µg/mL and 8 µg/mL) were chosen. Tubes were placed in a static incubator at 37 °C for 48 h; the time required for untreated wells to form biofilm. After 48 h, treated media was removed and wells were rinsed with 1 mL of PBS to remove planktonic cells. Following washing, 1 mL of nutrient-rich media (appropriate to each of the tested strains) was added to each tube. For each tube, a sterile cell scraper (Nunc Cell Scrapers, Thermo Scientific, Mississauga, ON, Canada) was used to carefully remove any adherent biofilm for 1 min, followed by vortexing the tube for an additional minute in order to help homogenize the solution. The resulting cell suspension was serially diluted, and viable colonies were enumerated by spreading on a nutrient agar plate using 4–6 sterile glass beads per plate. Plates were left at 37 °C for 18 h to allow colonies sufficient time to reach an easily observable size. Duplicate dilutions and plates were made for each tube. CFU was recorded. The experiment was carried out three independent times for each strain. 

#### 2.5.4. Enumeration of Viable Cells after Pre-Formed Biofilm Destruction

In order to investigate whether prevention or degradation of biofilm by peptide treatment impacted bacterial cell growth within the biofilm, an enumeration assay was carried out. The following protocol was developed using methods described in the work by Wilson et al. [48]. Briefly, in separate 15 mL culture tubes, 20 µL of overnight culture of either *E. coli*, *S. typhimurium*, or MRSA was added to 1 mL of minimal salt media (40m M NaOH∙ ∙7H_2_O; 22 mM KH_2_PO_4_; 18.68 M NH_4_CL; 740 mM NaCl; 2mM MgSO_4_; 0.1 mM CaCl_2_; 22 mM supplemented with d-Glucose). Tubes were placed in a static incubator at 37 °C for 48 h; the time required for untreated wells to form biofilm. After formation, minimal salt media was removed and tubes were treated with 1 mL of minimal salt media containing PG-1 or SynPG-1. Due to the laborious procedure, the study was limited to measuring cell viability for only two concentrations, basing the choice on the significant reduction of biomass from crystal violet staining. For instance, for *E. coli* significant biomass reduction was seen at 32 µg/mL and 64 µg/mL. These concentrations were chosen for the enumeration assay. Tubes were placed in a static incubator at 37 °C for 3 h. After 3 h, treated media was removed and wells were rinsed with 1 mL of PBS to remove planktonic cells. Following washing, 1 mL of nutrient-rich media (appropriate to each of the tested strains) was added to each tube. For each tube, a sterile cell scraper (Nunc Cell Scrapers, Thermo Scientific, Mississauga, ON, Canada) was used to carefully remove any adherent biofilm for 1 min, followed by vortexing the tube for an additional minute in order to help homogenize the solution. The resulting cell suspension was serially diluted, and viable colonies were enumerated by spreading on a nutrient agar plate using 4–6 sterile glass beads per plate. Plates were left at 37 °C for 18 h to allow colonies sufficient time to reach an easily observable size. Duplicate dilutions and plates were made for each tube. CFU was recorded. The experiment was carried out three independent times for each strain. 

### 2.6. LPS-Binding Assay 

The ability of each peptide to bind LPS was assessed by using a quantitative Chromogenic Limulus Amebocyte Lysate (LAL) assay (Pierce LAL Chromogenic LAL Endotoxin Quantitation Kit, Thermo Fisher Scientific, Mississauga, ON, Canada). Stocks of the peptide were prepared in endotoxin-free water, provided by the kit. Peptides were incubated with LPS from *E.coli O111:B4* (1 EU/mL) at concentrations of 10 µg/mL, 40 µg/mL, and 80 µg/mL in 0.6 mL sterile tubes on a heating block at 37 °C for 30 min to allow peptide binding to LPS. In a flat bottom, a non-pyrogenic 96-well plate (Costar Cell Culture Plates, Thermo Fisher Scientific, Mississauga, ON, Canada), maintained at 37 °C on a heating block with 50 µL of LPS-peptide solution, was dispensed into 2 replicate wells, followed by 50 µL of LAL reagent to each well. After 10 min of incubation, 100 µL of pre-warmed LAL Chromogenic substrate (acetyl-Ile-Glu-Ala-Arg-*p*-nitroanilide), solution was added to each well and incubated on the heating block for an additional 6 min. After incubation, 100 µL of stop reagent (25% acetic acid) was added to each well. The release of *p*-nitroanilide was detected through reading the absorbance at an OD of 410 nm. Absorbance readings from the negative control (LPS, without peptide) was compared with the absorbance reading of wells containing both LPS and peptide to calculate relative % LPS binding. 

### 2.7. In Vitro Cell Culture Assays

#### 2.7.1. Cell Culture Preparation 

A cell line, established from intestinal porcine enterocytes, isolated from the jejunum of a neonatal unsuckled piglet (IPEC-J2 cells, DSMZ), was grown in monolayer culture in Dulbecco’s modified Eagle’s medium (high glucose), supplemented with 10% fetal bovine serum (FBS) from Invitrogen, 100 IU/mL penicillin, and 100 g/mL streptomycin. Cultures were maintained in a 5% CO_2_ humidified atmosphere at 37 °C and passaged every 2–3 days, with detachment via the Trypsin-EDTA solution (Sigma).

#### 2.7.2. Cell Viability in the Presence of Antimicrobial Peptide

Cell viability was measured using the AlamarBlue cell viability reagent (Thermo Fisher Scientific, Mississauga, ON, Canada) and the instructions given in the protocol were followed. Briefly, IPEC-J2 cells were plated in a 96-well plate at a density of approximately 5000 cells/well. After 24 h, the serum was removed and the cells were treated for 2 h with SynPG-1 or PG-1, at concentrations ranging from 10 µg/mL to 100 µg/mL. After 2 h, alamar blue reagent was added to each well and incubated for another 1 h before fluorescence was measured at an OD of 570 nm. This assay was performed in the presence of 2% serum. This was conducted for three independent experiments, performed in triplicate. 

#### 2.7.3. Effect of Antimicrobial Peptide on In Vitro Cell Proliferation

IPEC-J2 cells were grown in 6-well plates (Corning, NY, USA) until 50% confluent in the medium, consisting of DMEM F-12 (1:1 Mixture of Dulbecco’s Modified Eagle Medium and Ham’s F-12 Medium), containing 5% FBS at 37 °C in 5% CO_2_, and were serum starved for 24 h. The cells were then incubated with serum-free DMEM-F12 in the presence or absence of either 1 µg/mL, 10 µg/mL, or 20 µg/mL of peptide. The treatments were incubated with the cells for 24 h. Cells were then trypsinized and enumerated using a Trypan Blue and hemocytometery method (BioRad T20 Automatic Cell Counter, Mississauga, ON, Canada). 

#### 2.7.4. Effect of Antimicrobial Peptide on In Vitro Cell Migration 

To evaluate migration of IPEC-J2 cells, 8-micron pore-sized cell culture transwell inserts were used (Millipore Inc, Temecula, CA, USA). A total of 1 × 10^5^ cells were plated in the upper inserts of the transwell and placed within 24-well plates, containing serum-free DMEM F-12 in the absence and presence of SynPG-1 or PG-1, at concentrations ranging from 5 µg/mL to 20 µg/mL. After incubation for 16 h, the cells were fixed with 4% (*w/v*) paraformaldehyde. Cells that did not migrate into the membrane were gently scraped off the upper surface of the transwell with a cotton swab. Migration was quantified by cell enumeration through Hoechst 33342 staining of cell nuclei (Thermo Fisher Scientific, Mississauga, ON, Canada). The results are represented as the relative fold increase in migrated cells when compared to the baseline migration of IPEC-J2 cells in serum-free media after 16 h. 

### 2.8. Statistical Analysis 

All assays were independently repeated, and results are shown with standard error. Statistical analysis was done using either one-way or two-way ANOVA, with either a post-hoc Dunnett or Tukey test. 

## 3. Results

### 3.1. Design of Novel Peptide SynPG-1

**Figure 1:**
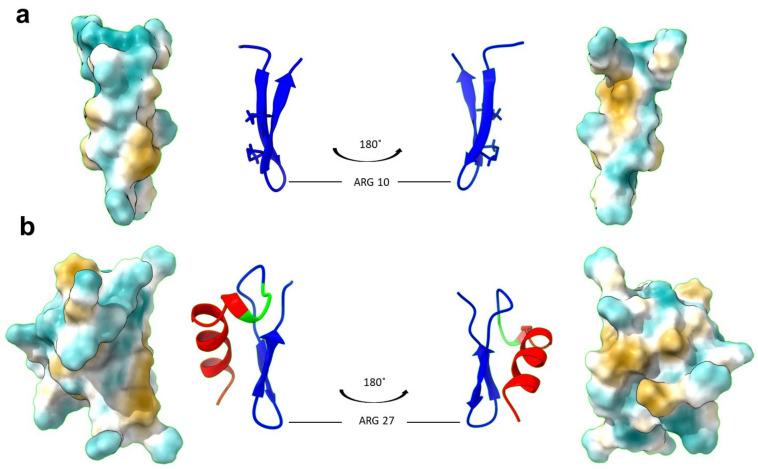
Refined 3D structure of the PG-1 and SynPG-1, generated using ChimeraX. (**a**) The 3D structure of PG-1 was obtained from the Protein Data Bank (PDB ID: 1PG1). Shown here are the front and back views of the secondary structure with disulfide bonds displayed, as well as the prediction of the hydrophobicity profile of the peptide surface, where blue indicates most hydrophilic regions and yellow indicates most hydrophobic regions. (**b**) The 3D structure of SynPG-1 was predicted using iterative threading ASSEmbly refinement (I-TASSER). Shown here are the front and back views of the secondary structure, with the Syn domain in red, the linker region in green, and the PG-1 domain in blue, as well as the prediction of the hydrophobicity profile of the peptide surface, where blue indicates most hydrophilic regions and yellow indicates most hydrophobic regions.

To investigate whether the addition of the LPS-binding domain, Syn, could improve the functional stability of the natural PG-1 peptide, we designed a hybrid peptide, SynPG-1. The Syn-domain sequence, shown in lowercase letters in Table 1, was joined at its C-terminal end to the N-terminal end of PG-1 (shown in capital letters) via a triple-glycine linker region (italicized). The amino acid sequence and chemical properties of both peptides are presented in Table 1. The net charge difference between the parent and hybrid peptides was +7. A 3D model of SynPG-1 was predicted through I-TASSER and visualized using ChimeraX. The comparison of the 3-dimensional structure of PG-1 (PDB ID: 1PG1) and the hybrid peptide can be seen in Figure 1.

### 3.2. Comparison of Antimicrobial Activity of PG-1 and SynPG-1

To test whether the addition of the LPS-binding domain, Syn, would enhance the peptide’s attraction to the bacterial surface and thereby increase its antimicrobial activity, a macrodilution antimicrobial assay was performed. The minimal bactericidal concentration (MBC) of PG-1 and SynPG-1 against planktonic cell growth of four multi-drug resistant (MDR) bacteria is displayed in Table 2. MBC was defined as the concentration that eliminated 99.9% of all colonies found on the negative control. SynPG-1 demonstrated greater antimicrobial activity against all tested strains, with MBC ranging from as low as 0.09 µM to 0.69 µM. Both peptides showed greater activity towards gram-positive strains than to gram-negative strains.

### 3.3. Comparison of Serum Stability of PG-1 and SynPG-1

**Figure 2:**
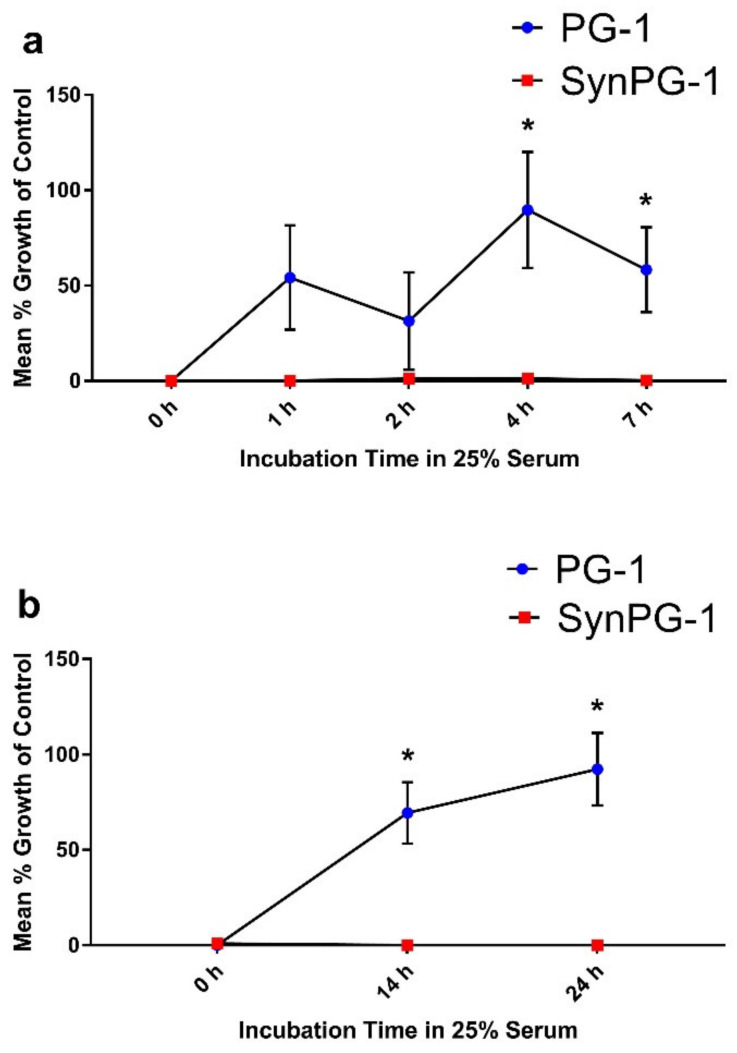
Serum stability of PG-1 and SynPG-1, represented by the mean % growth of control when tested against *E. coli* (**a**) after peptides were incubated for up to 7 h in 25% serum and (**b**) after peptides were incubated up to 24 h in 25% serum. All experiments were conducted three independent times. Peptides were both tested at a concentration 10 times their MBC (20 µg/mL). Stability was evaluated by comparing the mean growth of colonies on plates treated with peptides incubated in serum to colonies grown when treated without peptide. Data represent the mean ± SEM of three experiments. The asterisk represents *p* < 0.05 when compared with SynPG-1 by two-way ANOVA with a post hoc Tukey test.

Peptide stability to serum was investigated by conducting an antimicrobial assay against *E. coli* after peptides were incubated in 25% porcine serum for up to 7 or 24 h. As shown in Figure 2a, PG-1 maintained some antimicrobial activity for up to 2 h of serum incubation. After 4 h, its antimicrobial activity decreased significantly compared to the controls. On the other hand, SynPG-1 maintained strong antimicrobial activity even after 24 h of incubation in serum, indicated through a consistently low mean % growth, as compared to the controls seen in Figure 2b.

### 3.4. Comparison of Trypsin Stability of PG-1 and SynPG-1

**Figure 3:**
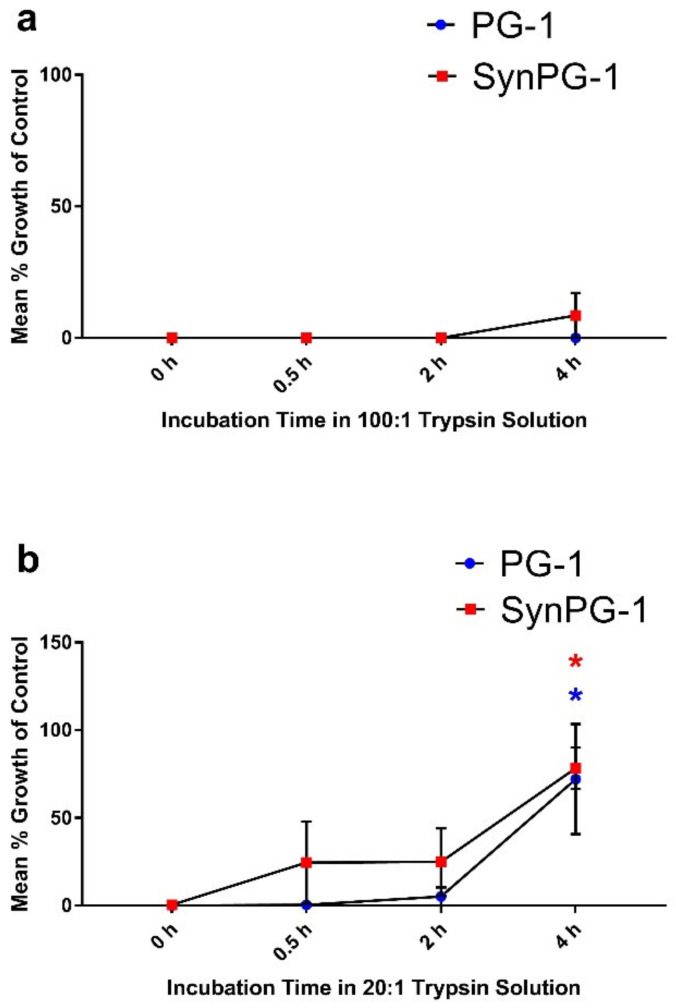
Stability of PG-1 and SynPG-1 against the trypsin enzyme, represented by the mean % bacterial growth of control tested against *E. coli* (**a**) after peptides were incubated at a ratio of 100:1 (peptide to trypsin) and (**b**) after peptides were incubated at a ratio of 20:1 (peptide to trypsin). Peptides were both tested at a concentration 10 times their MBC (20 µg/mL). Stability was evaluated by comparing the mean growth of colonies on plates treated with peptides incubated in trypsin to colonies grown when treated without peptide. All experiments were conducted three independent times. Data represent the mean ± SEM of three experiments. The red and blue asterisks represent *p* < 0.05 in SynPG-1 and PG-1, respectively, when compared to the control by two-way ANOVA with a post hoc Tukey test.

Trypsin is a major proteinase in the intestine. The impact of the trypsin enzyme on peptide stability was investigated by conducting an antimicrobial assay against *E. coli*. Stability was evaluated by comparing the mean growth of colonies on plates treated with peptides and incubated with trypsin to colonies grown when treated without peptide. The results of this assay are presented in Figure 3a as the mean % growth of control. As shown, both peptides maintained antimicrobial activity for up to 4 h of incubation with trypsin, when the concentration of peptide to the trypsin enzyme was 100:1. However, when the ratio of peptide to trypsin was reduced to 20:1, both peptides maintained activity for 2 h but saw increases in the mean % cell growth of the control after 4 h of incubation, as seen in Figure 3b.

### 3.5. PG-1 and SynPG-1 Stability in Porcine Digestive Fluids

**Figure 4:**
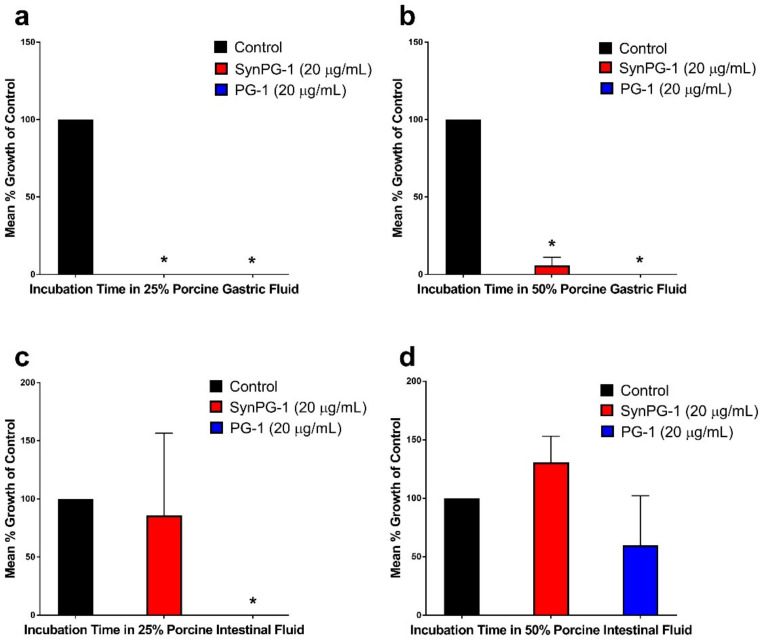
Stability of PG-1 and SynPG-1 in porcine digestive fluid, represented by the mean % bacterial growth of control when tested against *E. coli* after peptides were incubated in (**a**) 25% or (**b**) 50% gastric fluid and after peptides were incubated in (**c**) 25% or (**d**) 50% intestinal fluid. Peptides were both tested at a concentration 10 times their MBC (20 µg/mL). Stability was evaluated by comparing the mean growth of colonies on plates treated with peptides incubated in digestive fluids to colonies grown when treated without peptide. All experiments were conducted three independent times. The asterisks represent significant differences at *p* < 0.05 when compared to the control by one-way ANOVA with a post hoc Tukey test.

Peptide stability to porcine digestive fluids was investigated by monitoring the changes to antimicrobial activity against *E. coli* after peptides were kept in diluted solutions of either gastric fluid or intestinal fluid for 1 h. As shown in Figure 4a,b, both peptides maintained significant antimicrobial activity after incubation in solutions of 25% and 50% porcine gastric fluid. However, SynPG-1 appeared to have lower stability to porcine intestinal fluid, as seen in Figure 4c, and where the mean % growth of control increased for SynPG-1 but not PG-1. Both peptides lost significant activity after 1 h of incubation with 50% porcine intestinal fluid, as seen in Figure 4d. When intestinal fluid stability was repeated using simulated intestinal fluid (Figure S1), a similar pattern of activity was seen. 

### 3.6. Inhibition of Biofilm Formation by PG-1 and SynPG-1

**Figure 5:**
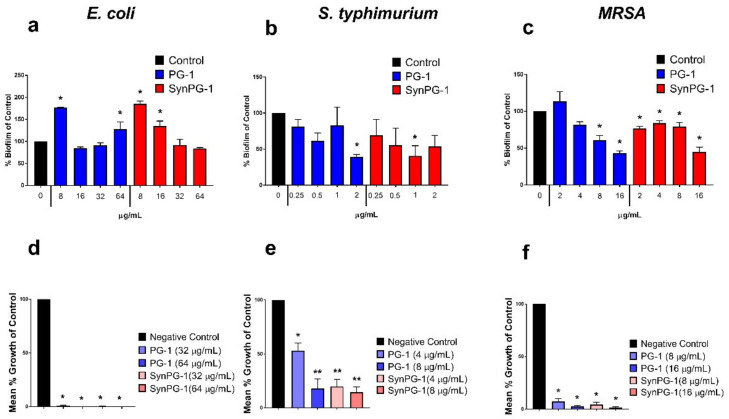
Effect of PG-1 and SynPG-1 on inhibition of biofilm formation of (**a**) *E. coli*, (**b**) *S. typhimurium*, and (**c**) MRSA, represented as the mean ± SEM of % biofilm of control of three independent experiments, where *n* = 6. The asterisks represent significant differences at *p* < 0.05 when compared to the control by two-way ANOVA with a post hoc Tukey test. The effect of PG-1 and SynPG-1 on the viability of (**d**) *E. coli*, (**e**) *S. typhimurium*, and (**f**) MRSA colonies, represented as the mean ± SEM of the relative mean % growth of control of three independent experiments performed in duplicate. The asterisks represent significant differences at *p* < 0.05 (*) or *p* < 0.01 (**), when compared through one-way ANOVA with a post hoc Tukey test.

The potential inhibitory effects of PG-1 and SynPG-1 on biofilm formation of three pathogenic bacterial strains was investigated through crystal violet biomass staining. Surprisingly, neither peptide was able to prevent biofilm formation of *E. coli*, instead showing significant promotion at certain concentrations (Figure 5a). Against *S. typhimurium,* significant inhibition was seen at 1 μg/mL for SynPG-1 and at 2 µg/mL for PG-1, as shown in Figure 5b. However, reduction in biofilm for this strain did not appear to act in a concentration-dependent manner, since significant inhibition was achieved for SynPG-1 at 1 µg/mL, but not 2 µg/mL. Against MRSA biofilms shown in Figure 5c, inhibition was seen at 2 µg/mL for SynPG-1 and 8 µg/mL for PG-1. In this instance, biofilm reduction appeared to be concentration dependent. SynPG-1 began to demonstrate a significant reduction in biofilm formation at a lower concentration than PG-1, which instead showed a significant promotion of biofilm formation at the same concentration.

To quantify the number of viable colonies remaining after biofilm inhibition, bacterial cells were enumerated after bacteria were given sufficient time to attempt biofilm formation in the presence of peptides. Concentration of peptide treatment was based off the effective concentrations in the crystal violet staining. Figure 5d–f show the mean % growth of control of bacterial cells growing inside peptide-treated biofilms versus non-treated bacteria. Both peptides substantially reduced the number of colonies growing within the biofilms of all three bacterial strains. SynPG-1 had a significantly lower reduction of mean % growth of control of *S. typhimurium* bacteria than PG-1 at 4 µg/mL.

### 3.7. Destruction of Pre-Formed Biofilm and Enumeration of Viable Cells

**Figure 6:**
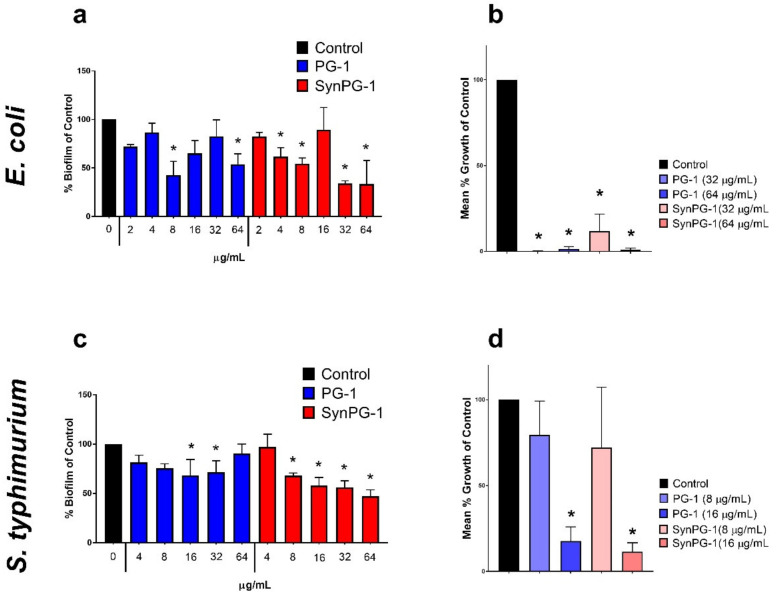
Effect of 3 h treatment with PG-1 and SynPG-1 on the destruction of pre-formed biofilm of (**a**) *E. coli* and (**c**) *S. typhimurium*, represented as the mean ± SEM of % biofilm of the control of three independent experiments, where *n* = 6. The asterisks represent significant differences at *p* < 0.05, when compared to the control by two-way ANOVA with a post hoc Tukey test. The effect of PG-1 and SynPG-1 on the viability of (**b**) *E. coli* and (**d**) *S. typhimurium* colonies, represented as the mean ± SEM of the relative mean % growth of control of three independent experiments performed in duplicate. The asterisks represent significant differences at *p* < 0.05, when compared with one-way ANOVA with a post hoc Tukey test.

The potential antibiofilm activity of PG-1 and SynPG-1 on pre-formed biofilms of three pathogenic bacterial strains was investigated using a crystal violet biomass staining method. Similar to their effect on the inhibition of biofilm production, again, peptides did not consistently demonstrate a concentration-dependent response. Against pre-formed *E. coli* biofilms, both peptides demonstrated significant disruption at 8 µg/mL and 64 µg/mL, but not at 16 µg/mL, as seen in Figure 6a. At 4 µg/mL and 32 µg/mL, only SynPG-1 had a significant reduction. Against pre-formed *S. typhimurium,* SynPg-1 began to show significant disruption at 8 µg/mL and both peptides at 16 µg/mL, as seen in Figure 6c. However, at 64 µg/mL, PG-1 did not demonstrate a significant reduction. Neither peptide showed any significant disruption or promotion of MRSA biofilms (data not shown, see Figure S2).

To quantify the number of viable colonies remaining inside biofilms after treatment, bacterial cells enumerated after pre-formed *E. coli* and *S. typhimurium* biofilms were treated with peptide. Concentration of peptide treatment was based off the lowest effective concentrations in the crystal violet staining. Figure 6b,e show the mean % growth of control of bacterial cells growing inside peptide-treated biofilms versus non-treated bacteria. Both peptides substantially reduced the number of colonies growing within the biofilms of both bacterial strains. 

### 3.8. LPS-Binding Ability

**Figure 7:**
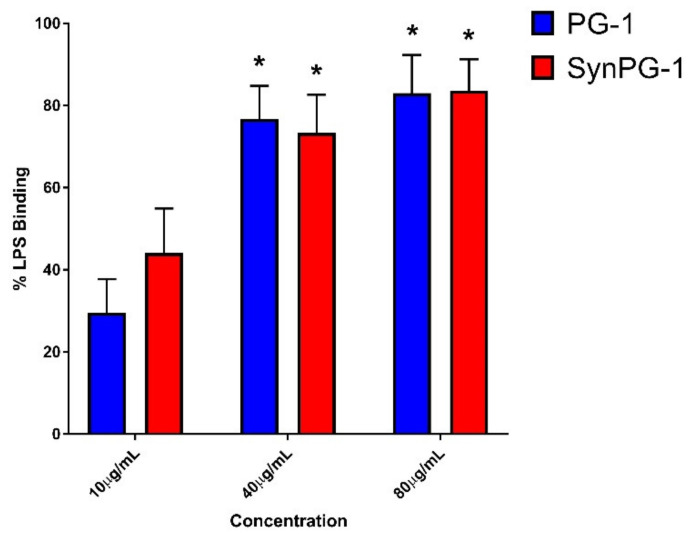
Comparison of the LPS binding ability between PG-1 and SynPG-1. The binding % was determined by measuring the ability to prevent LPS-induced activation of the LAL enzyme. Data represents the mean ± SEM of three independent experiments performed in duplicate. The asterisks represent a significant difference from PG-1 at 10 µg/mL at *p* < 0.05, as compared by two-way ANOVA with a post hoc Tukey test.

The LPS-binding activity of each peptide was measured by comparing the inhibition of the LPS-induced activation of the LAL enzyme in the presence or absence of peptide. These results are presented in Figure 7. Both peptides appeared to have potent LPS-binding activity that operated in a concentration-dependent manner. However, there was no significant difference between the two peptides at each concentration. For each peptide treatment, a lack of significant difference between 40 µg/mL and 80 µg/mL indicates a possible maximum binding saturation of peptide to LPS.

### 3.9. Cell Viability in the Presence of Antimicrobial Peptide 

**Figure 8:**
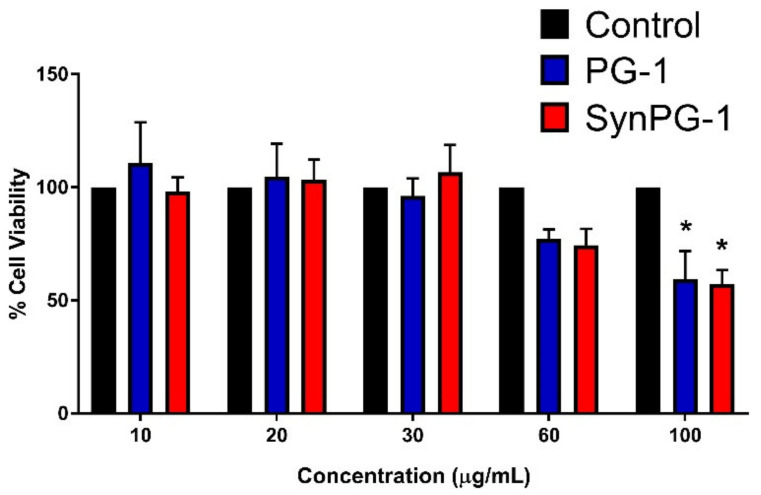
Comparing the cell viability of IPEC-J2 cells. Viability is represented as the % cell viability of cells in 2% serum after a 3 h incubation with peptide, relative to cells without peptide treatment. Data represents the mean ± SEM of three independent experiments, where *n* = 4. The asterisks represent a significant difference at *p* < 0.05, as compared to the control by two-way ANOVA with a post hoc Tukey test.

The effect of PG-1 and SynPG-1 on IPEC-J2 cell viability was measured to ensure that observed differences in cell response were not due to toxicity. As shown in Figure 8, neither peptide demonstrated significant reductions in cell viability until they reached 100 µg/mL. A comparison between peptides showed no difference in the impact on cell viability between PG-1 and SynPG-1. 

### 3.10. Effect of PG-1 and SynPG-1 on Cell Proliferation and Migration

**Figure 9:**
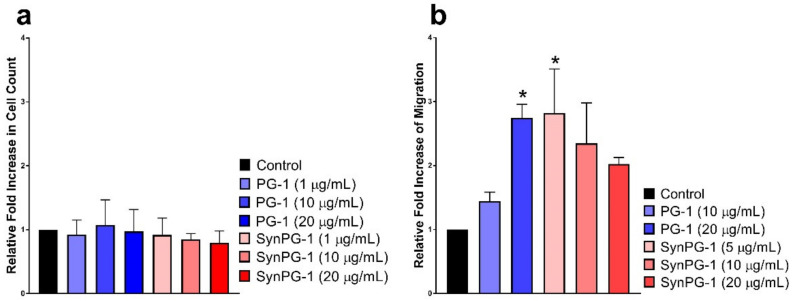
Comparing the effects of peptide treatment on stimulating (**a**) cell proliferation in IPEC-J2 cells after 24 h incubation in peptide treatment, represented by the relative fold increase in cell count, and (**b**) cell migration of IPEC-J2 after 16 h incubation is represented as the % cell viability of cells in 2% serum after a 3 h incubation with peptide, relative to cells without peptide treatment. Data represents the mean ± SEM of three independent experiments, where *n* = 4. The asterisks represent a significant difference at *p* < 0.05, as compared to the control by one-way ANOVA with a post hoc Dunnett test.

To study the potential mitogenic role of PG-1 and SynPG-1, proliferation was investigated by comparing the number of cells between cells treated with and without peptide. As shown in Figure 9a, no difference was seen in either of the antimicrobial peptide groups when compared to that of the control.

Meanwhile, the effect of the PG-1 and the hybrid peptide in stimulating cell migration was accessed through a transwell assay. As shown in Figure 9b, starting at 20 µg/mL, PG-1 increased cell migration significantly. The migration-inducing effect by SynPG-1 was observed at 5 µg/mL, although this activity was not significant at higher concentrations of the peptide.

## 4. Discussion

AMPs are an extraordinary source of novel antimicrobial agents, with mechanisms not exclusive to bactericidal activity, but also immune-stimulation [31], biofilm inhibition, and destruction [14]. Despite the potential they possess, AMP commercialization is challenged by their poor stability within the physiological environment [44]. Design approaches that improve stability, while preserving both the bactericidal activity and immunomodulatory activity of AMPs, is necessary for overcoming this challenge. 

That is why the present study is aimed at improving the activity and stability of a natural AMP through hybridization with an LPS-binding domain with stability-improving effects. Kim et al. designed the Syn-domain by comparing various sequences from natural peptides and proteins with known LPS-binding properties [35]. LPS is a major constituent present on the surface of Gram-negative bacteria and can therefore serve as a target for AMP binding [49]. When combined with AMPs, the novel peptide improved the antimicrobial activity towards gram-negative bacteria and had higher LPS-binding ability [35]. Additionally, in an unpublished study evaluating the stability of various hybrid peptides in serum, we found that the novel Syn-possessing peptide had the highest stability, indicating some potential protective effects through the addition of the Syn domain. The current study aims to test the hypothesis that the addition of the Syn domain could improve the potency of PG-1. 

The Syn-domain was designed with similar structural characteristics often used in creating AMPs. For instance, it has a net positive charge due to the inclusion of residues such as Arg and Lys [35], which is necessary for the electrostatic attraction of a peptide towards the negatively charged bacterial membrane [50,51]. Secondly, it possesses high hydrophobic content due to the inclusion of residues such as Leu, Ile, and Trp [35], which is necessary for insertion into the lipid bilayer and eventual membrane disruption [50,51]. Possessing these two design characteristics, the Syn domain was reported to demonstrate some antimicrobial activity alone [35]. With this same principle in mind, it was theorized that since SynPG-1 had higher hydrophobic content and a greater net positive charge than PG-1, it would have greater bactericidal activity. 

Although SynPG-1 had a predicted molecular weight approximately double that of PG-1 (see Table 2), in the interest of providing a more conservative comparison and to better compare the results to future potential animal experiments, we chose to evaluate the two peptides based on equal mass concentration. Thus, when making a comparison based on a mass to volume ratio (µg/mL), both peptides displayed the same level of antimicrobial activity. However, when comparing molar concentration (µM), SynPG-1 consistently displayed an MBC 2-fold lower than that of PG-1, as shown in Table 2.

Similarly, Kim et al. reported a synergistic effect in their hybrid peptide’s activity towards planktonic bacteria [35]. However, the difference reported was much higher than the one observed in this study, with as much as a 32-fold difference in minimal inhibitory concentration between the parent and hybrid peptide for gram-negative strains [35]. The lack of substantial increase in activity between PG-1 and SynPG-1 indicates that other factors, aside from total cationicity and hydrophobicity, may play an important role in governing the relationship between AMP’s charge and bactericidal killing. For instance, Bolintineanu et al., who studied the pore forming mechanism by which PG-1 administers its antimicrobial activity, demonstrated how a threshold of sufficient pore formation must be reached by a peptide before bacterial death can be achieved [52]. The process of pore formation is thought to be facilitated through hydrogen bonds between PG-1 monomers [53], and insertion into the membrane occurs only after sufficient oligomerization [54]. Therefore, although the addition of the Syn-domain may improve the adherence of the peptide to the cell membrane, the resulting antimicrobial activity may still be governed by the peptide–to–peptide interaction of the PG-1 domain within SynPG-1. More investigation is needed to understand how hybridization of the Syn domain and PG-1 impact peptide oligomerization and subsequent antimicrobial activity. 

While numerous publications have aimed to study the structural properties of AMPs in their relation to membrane disruption and have provided valuable insight on this complex interaction [55,56,57,58,59,60], more consideration needs to be given to the impact of the physiological environment on AMP activity. For this reason, the primary goal of this study was to evaluate the functional stability of the novel designed AMP by evaluating bactericidal activity after pre-incubation in physiological fluids. Previously, many publications have sited PG-1 as being resistant to serum. However, this observation appears to be an extrapolation of PG-1′s resistance to saline environments [61], rather than a direct observation. While it is important to test antimicrobial activity with salt, it is important to recognize how binding by serum proteins, such as albumin, can inhibit AMP activity [62]. In this study, the impact of serum on PG-1 caused an immediate reduction in its antimicrobial activity, following 1–2 h of pre-incubation. However, significant loss of activity was not seen until 4 h of pre-incubation. In contrast, SynPG-1 was found to retain its activity after pre-incubation in porcine serum for at least 24 h. Recently, Yang et al. ran a similar experiment for their design of hybridized insect cecropin A and Fowlicidin-2, which retained high antimicrobial activity after 2 h of pre-incubation in 12.5%, 25%, and 50% human serum [63]. Similarly, a truncated analogue of the snake venom cathelicidin-like peptide crotalicidin has been reported as having exceptionally high stability of human serum for up to 12 h of incubation [64]. Perez-Peinado et al. studied synthetic analog variants of this peptide to try to understand the reason for its remarkably slow degradation within serum. They concluded that the location of the alpha-helix domain at the N-terminal end, when followed by a hydrophobic domain, was a contributing structural factor in the slow degradation of the peptide in serum [65]. 

A possible explanation for SynPG-1′s high resistance to serum inactivation could be due to self-assembly [66]. Within aqueous solutions, the amphipathic structure of AMPs can promote self-assembly of their monomers into aggregates, where hydrophobic residues are located inside the aggregate body and hydrophilic are outside [66]. It has been suggested that this aggregation can help protect the AMP from cleavage if vulnerable sequence motifs are located within the hydrophobic region [66]. Although more research is necessary to explain SynPG’s stability within serum, these initial findings highlight the hybrid peptide’s potential for treatment of topical and systemic infections.

However, this remarkable serum stability did not entirely translate to stability within solutions of gastric and intestinal fluid. While both peptides were able to withstand 1h of pre-incubation in 25% and 50% porcine gastric fluid, respectively, a greater susceptibility to porcine intestinal fluid was evident. One limitation to this experiment was that we were not able to directly quantify the concentration of enzymes in the collected fluid. However, a follow-up study using simulated intestinal fluid, containing a known concentration of trypsin enzyme, suggested that the reduction in activity was likely due to degradation by a trypsin enzyme (See Figure S1). Previously, our group has investigated the potential of PG-1 as treatment for colitis and have observed its therapeutic ability to positively influence intestinal tissue repair [67] and mediate inflammatory markers in experimentally-infected mice [68]. The trypsin and gastrointestinal fluid stability results of this current study suggest that there is potential room for improving PG-1 stability towards endogenous proteases and that this improvement may result in increasing its therapeutic potential.

Although many have studied the antimicrobial potential of PG-1, its anti-biofilm properties have only been tested against a few species, including *Streptococcus mutans* and *Acinetobacter baumanni* [69,70]. Since the composition of the extracellular polymeric substance (EPS) of a biofilm can vary widely from species to species [71], further investigation into the PG-1′s spectrum of antibiofilm activity is necessary. To do so, we evaluated the effect of PG-1 and SynPG-1 on the disruption and formation of biofilms from three pathogenic MDR bacteria and measured the remaining viable pathogens. Within our study, the marginal reduction in the biomass observed through crystal violet staining, coupled with the substantial reduction in viable bacteria within the biofilm, suggest that both PG-1 and SynPG-1 have a mechanism that allows them to penetrate the EPS and facilitate bactericidal killing. Liu et al. has reported PG-1′s anti-biofilm activity against *S. mutans*. Their study revealed that green fluorescent protein-tagged (GFP)PG-1 had little effect in degrading the EPS, but had a marginal reduction of viable cells when treated with 10 µg/mL of peptide for 1 h [69]. Additionally, they noted that the combination of EPS-degrading enzymes with GFP-PG-1 had a synergistic effect in further lowering cell count. In contrast, Morroni et al. recently reported finding no antibiofilm activity in PG-1 when tested alone or with an antibiotic against *A. baumanni* biofilm [70]. However, their study did not enumerate bacteria from within the biofilm and they based their assessment only on the reduction in crystal violet staining.

The susceptibility of bacteria within biofilm forming conditions towards PG-1 and SynPG-1 may differ to their planktonic counterparts. This is evident through the fact that biofilm formation was not entirely inhibited or even promoted by the presence of peptide at lower concentrations. Viable biofilm colonies still remained even after incubation with peptides at concentrations 16 times higher than the MBC of their planktonic counterparts. Other authors have also noted a lack of correlation between Minimal Inhibitory Concentration (MIC) and MBC values and effective biofilm reduction [72], some even reporting anti-biofilm activity at concentrations lower than the MBC [11]. A possible explanation proposed for this phenomena could be related to the fact that bacteria within biofilms operate at a lower metabolic status than their planktonic counterparts [2] and have been observed to be genotypically and phenotypically distinct [73]. However, how these changes can impact a peptide’s antimicrobial activity is unclear. 

Alternatively, the difference between the MBC of planktonic bacteria and their biofilm-forming counterparts may be due to a loss of peptide after interaction with EPS constituents. Some AMPs have been known to become trapped within the EPS due to the strong attraction between negatively charged EPS components and the cationic charge of AMPs, thus making them devoid of any anti-biofilm activity [72] or degraded by biofilm enzymes. Therefore, in the process of translocating through or disrupting the EPS, some peptide may have been lost in the process. This may also account for the concentration-independent response seen in the crystal violet assay. Additionally, the promoting biofilm formation was at lower concentrations (Figure 5a,c). On the other hand, the difference may also be associated with the fact that bacteria within biofilms adhere to one another, forming multiple layers [13]. Thus, peptide may simply have limited access to the bacteria found further within the multilayered colonies. Further investigation is necessary for understanding AMP interactions with bacterial biofilms.

Seeing as the Syn-domain was designed to be an LPS binding domain [35], we aimed to compare the binding ability between our hybrid peptide. With the addition of the Syn-domain to their AMP, Kim et al. had reported to achieve almost 100% LPS binding [35]. To our surprise, the addition of the LPS-binding domain Syn to PG-1 did not significantly improve LPS binding in our comparison. Peptide binding to aqueous LPS occurs through similar mechanisms of electrostatic attraction as for binding to surface-embedded LPS [74]. It is unclear as to why no substantial increase in binding was observed. In contrast, Mohanram et al. achieved more than 75% LPS binding in a hybrid peptide possessing unique synthetic beta-boomerang motif with LPS binding activity, where the parent peptide had limited LPS binding [33]. Consistent to the report by others [75], we observed PG-1 binds LPS in vitro. The lack of improvement in LPS binding by the addition of the Syn domain is surprising. 

Previously, our lab explored effects of PG-1 on stimulating cell migration on the IPEC-J2 cell line [19]. In the current study, we assessed whether the hybrid peptide, SynPG-1, retains this function. The current study observed a significant increase in cell migration of cells by PG-1 (20 µg/mL) and SynPG-1 (5 µg/mL), respectively, suggesting that the addition of the Syn domain to the antimicrobial peptide retains this stimulating function of cell migration and is thus a potential tissue repair function. 

## 5. Conclusions

In summary, this study showed that the hybrid design of the AMP SynPG-1 substantially improved the serum stability of PG-1. The improved stability of the peptide, coupled with its persistent antimicrobial activity against both planktonic and biofilm multi-drug resistant bacteria and high LPS binding, suggests that this modified AMP is an improved candidate for clinical topical and systemic administration application.

## Figures and Tables

**Table 1:** The amino acid sequence of the studied peptides and their predicted properties.

Peptide	Amino Acid Sequence^1^	Amino Acid	Net Charge	Molecular Weight
Protegrin-1 (PG-1)	**R**GG**R**LCYC**RRR**FCVCVG**R**	18	+6	2160.63
Syn-Protegrin-1 (SynPG-1)	gl**rr**ll**rk**i**r**g**r**w**k***GGG***R**GG**R**LCYC**RRR**FCVCVG**R**	35	+13	4122.02

^1^**Boldface** residues represent basic amino acids and underlined residues represent hydrophobic amino acids.

**Table 2:** The minimal bactericidal concentration (MBC) of peptides against planktonic bacterial cells.

Bacteria	MBC μg/mL (µM)	
	PG-1	SynPG-1
*Escherichia coli*	2 (0.67)	2 (0.35)
*Salmonella typhimurium*	4 (1.34)	4 (0.69)
MRSA ^1^	0.5 (0.18)	0.5 (0.09)
MRSP ^2^	0.5 (0.18)	0.5 (0.09)

^1^ Methicillin-resistant *Staphylococcus aureus*. ^2^ Methicillin-resistant *Staphylococcus pseudointermedius*.

## Supplementary Materials

The following are available online at https://www.mdpi.com/2218-273X/10/7/1014/s1, Figure S1: PG-1 and SynPG-1 stability in simulated intestinal fluid; Figure S2: MRSA; Table S1: The antibiogram of clinical isolates used in this study.
