# Supplementary material for: The Addition of a Synthetic LPS-Targeting Domain Improves Serum Stability While Maintaining Antimicrobial, Antibiofilm, and Cell Stimulating Properties of an Antimicrobial Peptide
Source: Biomolecules. 2020 Jul 8;10(7):1014. doi: 10.3390/biom10071014 (PMC7407491; doi:10.3390/biom10071014)
Supplement: Supplementary file 1 [file biomolecules-10-01014-s001.pdf]

Table S1. The antibiogram of clinical isolates used in this study.

Antibiotic	*Escherichia coli*		*Methicillin Resistant Staphylococcus aureus*		*Methicillin Resistant Staphylococcus pseudointermedius*	
	Interpretation[1]	MIC[2]	Interpretation	MIC	Interpretation	MIC
**Amikacin**	/	/	S (18 mm)	9.5	/	/
**Amoxicillin-clavulanate**	R (8 mm[3])	>64	R (16 mm)	14	R (24 mm)	2.9
**Ampicillin**	R (6 mm)	>46	R (11 mm)	19	R (13 mm)	12
**Carbenicillin**	/	/	R (15 mm)	N/A	/	/
**Cefixime**	/	/	R (6 mm)	>26	/	/
**Cefovecin**	R (6 mm)	N/A	/	/	R (21 mm)	N/A
**Cefoxitin**	R (6 mm)	>69	/	/	R (24 mm)	N/A
**Ceftazidime**	/	/	R (13 mm)	41	/	/
**Ceftiofur**	/	/	R (19 mm)	3.9	/	/
**Cephalothin**	R (6 mm)	>101	R (25 mm)	2.2	R (27 mm)	1.4
**Chloramphenicol**	/	/	S (26 mm)	4.5	/	/
**Clindamycin**	/	/	R (6 mm)	>10	R (7 mm)	>10
**Difloxacin**	/	/	S (30 mm)	<=0.07	/	/
**Enrofloxacin**	R (7 mm)	>18	S (29 mm)	<=0.099	R (6 mm)	>19
**Gentamicin**	S (18 mm)	2.6	R (9 mm)	31	I (14 mm)	7.9
**Imipenem**	/	/	R (39 mm)	<=0.56	/	/
**Marbofloxacin**	R (6 mm)	N/A	S (27 mm)	N/A	R (6 mm)	N/A
**Orbifloxacin**	R (6 mm)	>40	S (27 mm)	0.25	R (6 mm)	>40
**Oxacillin**	/	/	R(6 mm)	N/A	R (14 mm)	1.8
**Piperacillin**	/	/	R (12 mm)	N/A	/	/
**Polymixin-B**	/	/	R (9 mm)	/	/	/
**Tetracycline**	R (13 mm)	16	S (31 mm)	0.42	R (8 mm)	>33
**Ticarcillin**	/	/	R (14 mm)	N/A	/	/
**Titracillin-clavulanate**	/	/	R (15 mm)	N/A	/	/
**Trimethoprim-sulfamethoxazole**	R (6 mm)	>10	S (31 mm)	<=4.1	R (6 mm)	>10

[1] Where R represents "resistant", I represents "intermediate" and S represents "sensitive".

[2] MIC represents the minimal inhibitory concentration of the antibiotic in mcg/mL.

[3] mm represents the diameter zone of inhibition against antibiotic disc.

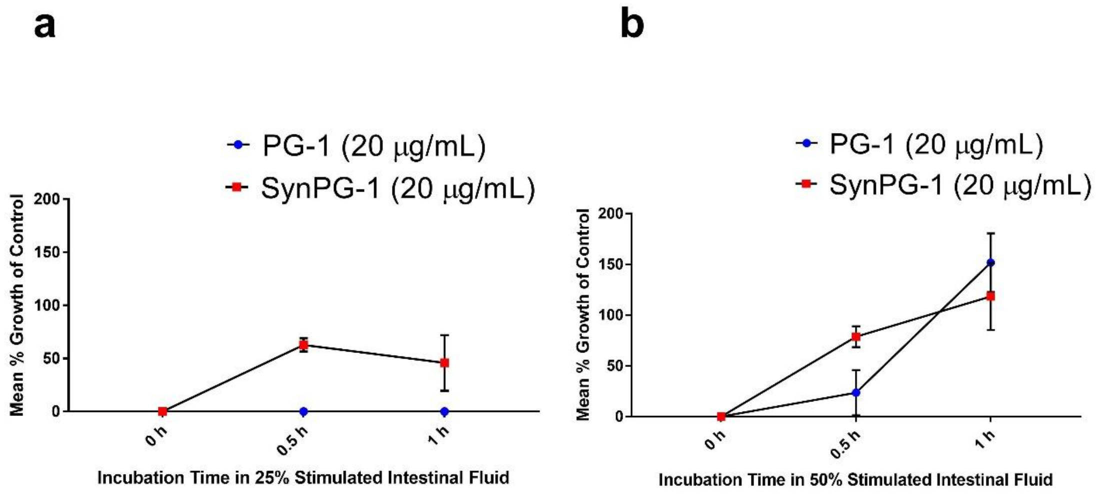

Figure S1. PG-1 and SynPG-1 stability in simulated intestinal fluid.

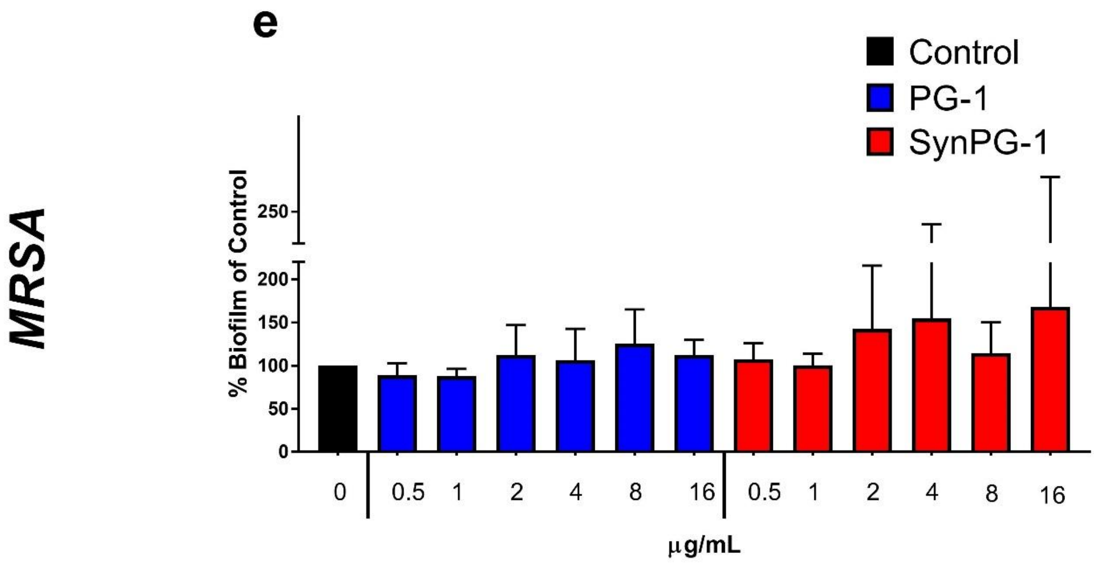

Figure S2. MRSA.
